# The role of type 2 diabetes in the association between habitual glucosamine use and dementia: a prospective cohort study

**DOI:** 10.1186/s13195-022-01137-x

**Published:** 2022-12-13

**Authors:** Chenjie Xu, Yabing Hou, Xuexian Fang, Hongxi Yang, Zhi Cao

**Affiliations:** 1 School of Public Health, Hangzhou Normal University, Hangzhou, China; 2 Yanjing Medical College, Capital Medical University, Beijing, China; 3 School of Basic Medical Sciences, Tianjin Medical University, Tianjin, China; 4 School of Public Health, Zhejiang University School of Medicine, No.866, Yuhangtang Road, Xihu District, Hangzhou, 310058 China

**Keywords:** Glucosamine, Dementia, Type 2 diabetes, *APOE* genotype

## Abstract

**Background:**

Growing evidence has showed an association between habitual glucosamine use and type 2 diabetes (T2D). However, the effect of habitual glucosamine use on risk of dementia remains poorly understood. Our study aimed to examine the association between glucosamine use and risk of dementia and further to identify the mediating role of T2D in the association.

**Methods:**

A total of 495,942 participants from UK Biobank who completed a questionnaire on habitual glucosamine use were included at baseline (2006–2010) and then followed up for incidence of dementia until 2020. Cox proportional hazard regressions were performed to calculate hazard ratios (HRs) and 95% confidence intervals (CIs) for incident dementia. Markov multi-state models were used to explore the role of incidence of T2D during the follow-up in the association.

**Results:**

Overall, 18.80% of the participants reported habitual use of glucosamine at baseline. A total of 6831 dementia events were recorded during a median follow-up of 11 years. In fully adjusted models, habitual glucosamine use was associated with a significantly lower risk of dementia (HR = 0.87, 95% CI: 0.82–0.93). Multi-state models showed that the association between glucosamine use and dementia was mediated by the incidence of T2D during the follow-up (HR of dementia without T2D: 0.92, 95% CI: 0.86–0.99; HR of post-T2D dementia: 0.79, 95% CI: 0.67–0.93).

**Conclusions:**

Our findings reveal that habitual use of glucosamine supplement is associated with a lower risk of dementia, which might be explained by incidence of T2D.

**Supplementary Information:**

The online version contains supplementary material available at 10.1186/s13195-022-01137-x.

## Background

It is estimated that around 50 million people are diagnosed with dementia worldwide [1]. The development of dementia is influenced by genetic, lifestyle, and medical risk factors [2]. So far, dementia can be neither cured nor reversed by available pharmacological interventions. However, a recent report from the Lancet Commission declared that 40% of dementia could be prevented or delayed if some major risk factors would be modified, including dietary factors [3]. Emerging evidence showed that diet quality and dietary nutrients appear to play important roles in preventing cognitive decline [4, 5]. For instance, dietary intakes of vitamin D, omega-3, and omega-6 fatty acids were found to be inversely associated with cognitive decline in the elderly [6, 7].

As a common nutritional supplement, glucosamine is widely recommended to use for managing osteoarthritis or joint pain in European [8, 9]. Meanwhile, it is regarded as one of the popular nutraceuticals in the USA and Australia [10, 11]. In vitro and in vivo experiments have revealed that glucosamine exerts beneficial effects by suppressing inflammation and antioxidant activities [12]. Brain and neural cells are highly sensitive to oxidative stress, which may cause profound cell damages and provoke neurodegenerative disorders [13]. Nevertheless, epidemiological evidence about glucosamine use and dementia is unclear to date. Previous longitudinal studies suggested that glucosamine use was associated with lower risk of type 2 diabetes (T2D) [14]. We hypothesized that T2D may play an essential role in the association between habitual glucosamine use and dementia. In this large-scale cohort study in UK Biobank, we aimed to investigate the association between habitual glucosamine use and dementia and explore the roles of T2D in the association.

## Methods

### Study population

UK Biobank is a large-scale biomedical study designed to contribute to modern medicine and improve public health [15, 16]. Participants aged 37 to 73 years were recruited across 22 assessment centers in the UK during 2006 to 2010. Details of its study design have been reported previously [17]. Participants were invited to provide information on lifestyle and medical history, take part in physical measurements, and provide biological samples regularly in the assessment centers at baseline. At the same time, health-relevant records of them would be linked to the UK National Health Service System throughout the study. The North West Multi-Centre Research Ethics Committee (reference 11/NW/0382) had approved the UK Biobank as a Research Tissue Bank.

We excluded participants (1) with prevalent dementia at baseline, (2) who did not complete the self-report assessments of habitual glucosamine use, and (3) who requested to be removed from the UK Biobank dataset. Our final analysis samples included 495,942 participants for disease outcomes. Flowchart of participant enrolment can be seen in Supplementary Fig. 1.

### Assessment of glucosamine use

At the assessment centers, participants were asked “Do you regularly take any of the following?” through a touchscreen questionnaire. Of all options, six dietary supplements could be selected, including glucosamine use. They can choose more than one answer in the questionnaire. We defined the use of glucosamine as: “0 = no” and “1 = yes”.

### Ascertainment of outcomes

The primary outcome in the current study was the incidence of dementia, and the secondary outcomes included the incidence of Alzheimer’s disease (AD) and vascular dementia (VD). Record linkage containing admissions and diagnoses information was linked to the Hospital Episode Statistics, Scottish Morbidity Record data, and the Patient Episode Database. Dementia events during the follow-up were ascertained from hospital inpatient records according to the International Classification of Disease version 10 (ICD-10) codes: F00-F03, and G30-G31 (Supplementary Table 1) [18]. Follow-up of this study was censored at the date of incident dementia, death, or the end of follow-up (September 30, 2020), whichever occurred first.

### Covariates

We collected the potential confounders at baseline, including age, sex (female, male), ethnicity (White, Black, south Asian, and mixed background), socioeconomic status (Townsend deprivation index), education attainment (college or university degree, professional qualifications, and others), body mass index (BMI, calculated as weight divided by the square of height, kg/m^2^), self-reported smoking status (never, previous, current), alcohol consumption (g/day), total cholesterol (mmol/L), family history of dementia (no or yes), aspirin use (no or yes), and mineral and vitamin supplements use (vitamin A, B, C, D, E; multivitamin; or folic acid).

The Townsend deprivation index was assigned as a continuous measure on the basis of postal codes, which were derived from census data on housing, employment, social class, and car availability; a higher index indicated more deprivation [19]. A healthy diet score was also summarized by using the following food categories: ≥ 4.5 servings total fruit and vegetable intake consumption per week, ≥ 2 fish intake per week, ≤ 2 times intake of processed meat per week, and ≤ 5 times red meat intake per week [20]. A healthy diet was ascertained if an individual met at least two healthy food items, as described in the previous study. Prevalent hypertension was defined as systolic blood pressure ≥ 140 mm Hg, or diastolic blood pressure ≥ 90 mm Hg, or reported use of antihypertensive drugs. In the main analyses, missing information on covariables was coded as a missing indicator category for categorical variables such as smoking status and with mean values for continuous variables. Detailed information of the missing variables can be seen in Table 1.

**Table 1:** Baseline characteristic of participants stratified by glucosamine use

Characteristics	Overall (***n***=495,942)	Glucosamine non-users (***n***=401,444)	Glucosamine users (***n***=94,498)	***P*** value
Age (years), mean (SD)	56.54 (8.09)	55.94 (8.20)	59.08 (7.07)	<0.001
Female	270,044 (54.45)	210,902 (52.54)	59,142 (62.59)	<0.001
Ethnicity				<0.001
White	467,865 (94.34)	377,256 (93.97)	90,609 (95.88)	
Black	7906 (1.59)	6866 (1.71)	1040 (1.10)	
South Asian	11,123 (2.24)	9627 (2.40)	1496 (1.58)	
Mixed background	7344 (1.48)	6275 (1.56)	1069 (1.913)	
Missing	1704 (0.34)	1420 (0.35)	284 (0.30)	
Townsend deprivation index				<0.001
1 (Least deprived)	99,244 (20.01)	77,476 (19.30)	21,768 (23.04)	
2	99,620 (20.09)	78,450 (19.54)	21,170 (22.40)	
3	99,304 (20.02)	79,407b (19.78)	19,897 (21.06)	
4	98,711 (19.90)	80,915 (20.16)	17,796 (18.83)	
5 (Most deprived)	99,063 (19.97)	85,196 (21.22)	13,867 (14.67)	
Missing	618 (0.12)	538 (0.13)	80 (0.08)	
Education attainment				<0.001
College or university degree	160,792 (32.42)	129,576 (32.28)	31,216 (33.03)	
Professional qualifications	245,272 (49.46)	197,114 (49.10)	48,158 (50.96)	
Others	84,679 (17.07)	70,392 (17.53)	14,287 (15.12)	
Missing	5199 (1.05)	4362 (1.09)	837 (0.89)	
Body mass index (kg/m^2^), mean (SD)	27.43 (4.79)	27.45 (4.82)	27.36 (4.64)	<0.001
Smoking status				<0.001
Never	270,649 (54.57)	218,693 (54.48)	51,956 (54.98)	
Previous	171,298 (34.54)	135,197 (33.68)	36,101 (38.20)	
Current	52,132 (10.51)	46,012 (11.46)	6120 (6.48)	
Missing	1863 (0.38)	1542 (0.38)	321 (0.34)	
Alcohol consumption (g/day), mean (SD)	14.54 (18.25)	14.66 (18.66)	14.05 (16.37)	<0.001
Healthy diet^a^				<0.001
Yes	249,104 (50.23)	192,905 (48.05)	56,199 (59.47)	
No	242,069 (48.81)	204,286 (50.89)	37,783 (39.98)	
Missing	4769 (0.96)	4253 (1.06)	516 (0.55)	
*APOE ε*4 carrier	137,781 (27.78)	111,708 (27.83)	26,073 (27.59)	<0.001
Carrier	137,781 (27.78)	111,708 (27.83)	26,073 (27.59)	
Non-carrier	346,783 (69.92)	280,342 (69.83)	66,441 (70.31)	
Missing	11,378 (2.29)	9394 (2.34)	1984 (2.10)	<0.001
Family history of dementia	65,161 (13.14)	50,620 (12.61)	14,541 (15.39)	<0.001
Total cholesterol (mean, mmol/L), mean (SD)	5.69 (1.11)	5.67 (1.11)	5.81 (1.09)	<0.001
Mineral and vitamin supplements use	157,450 (31.75)	104,927 (26.14)	52,523 (55.58)	<0.001
Aspirin use	69,318 (13.698)	56,018 (13.95)	13,300 (14.07)	0.338
Hypertension	345,726 (69.71)	278,411 (69.35)	67,315 (71.23)	<0.001
Arthritis	52,214 (10.53)	32,973 (8.21)	19,241 (20.36)	<0.001

Values are numbers (percentages) unless stated otherwise

The number of missing data for BMI is 2579 (0.52%), alcohol consumption is 427 (0.09%), total cholesterol is 28,559 (5.76%)

^a^Healthy diet score was summarized by using the following food categories: 20 ≥ 4.5 servings total fruit and vegetable intake consumption per week, ≥ 2 fish intake per week, ≤ 2 times intake of processed meat per week, and ≤ 5 times red meat intake per week. A healthy diet was defined as a diet score of 2 or more

### Statistical analyses

We used the mean and standard deviation (SD) to describe continuous variables with normal distribution, and numbers (percentages) to describe categorical variables. Baseline characteristics were compared based on whether participants used or did not use glucosamine. We used the *χ*^2^ test for categorical variables, or analysis of variance or Mann-Whitney *U* test for continuous variables, as appropriate.

Cox proportional hazards models with age as timescale were used to calculate hazard ratios (HRs) and 95% confidence intervals (CIs) of dementia. The proportional hazards assumptions were tested based on Schoenfeld residuals [21]. Participants who reported themselves as glucosamine non-users were considered as the reference group. Three models were estimated in the following analyses. Model 1 was adjusted for age (timescale) and sex. Model 2 was additionally adjusted for ethnicity, BMI, education attainment, Townsend deprivation index, smoking status, alcohol consumption, family history of dementia, hypertension, arthritis, total cholesterol, healthy diet, and *APOE* genotype. Model 3 was finally adjusted for ethnicity, BMI, education attainment, Townsend deprivation index, smoking status, alcohol consumption, family history of dementia, hypertension, arthritis, total cholesterol, *APOE* genotype, healthy diet, use of mineral and vitamin supplements, and use of aspirin.

We assessed the additive- and multiplicative-scale interaction measures to examine the interaction effect of glucosamine use and *APOE ε*4 genotype on the risk of dementia onset. We calculated the attributable proportion due to interaction (AP), relative excess risk due to interaction (RERI), and synergy index (SI) to derive the additive interaction. Above mentioned indices were used to assess whether the risk due to having both exposures is greater than the sum of the risks due to each condition. The multiplicative-scale interaction has been widely used to examine the interaction effects by identifying whether the risk due to having both exposures is greater than the product of the risks due to each exposure alone [22].

In subsequent analyses, Markov multi-state models were used to explore the role of T2D over the follow-up in the association between glucosamine use and risk of dementia. The Markov multi-state models were semi-parametric models which could facilitate data preparation and flexible estimation of different types of covariate effects in Cox regression models [23]. It is a useful way of describing a process in which an individual moves through a series of states in continuous time. These models allow simultaneous estimation of the association of glucosamine use with risk of incident T2D over the follow-up (Transition 1), the association of glucosamine use with risk of dementia without incident T2D over the follow-up (Transition 2), and the association of glucosamine use with risk of dementia following diagnosis of T2D (Transition 3). The models were adjusted for the covariates mentioned above.

Several sensitivity analyses were finally performed to confirm the robustness of our results. First, those who developed dementia events within 2 years of follow-up were excluded to reduce the possibility of spurious association due to reverse causation. Second, we conducted the complete-case analyses and multiple imputation using chained equations with 5 imputations. Third, the competing risk of death on the association between glucosamine use and dementia was investigated using flexible parametric competing risk model [24]. Finally, we also calculated the *E*-value to evaluate the risk of unmeasured confounding. The *E*-value estimates the minimum strength that an unmeasured confounding variable would have to have to nullify the observed association between glucosamine use and dementia while considering all other measured covariates [25].

We used Stata to conduct all the statistical analyses (version 15, StataCorp). The statistical significance was set as *P* < 0.05 (two-sided test). Bonferroni correction (significance level 0.05/16 = 0.003) was conservatively corrected when we tested the effect modifications.

## Results

### Baseline characteristics of population

A total of 495,942 participants were included in the analyses. Table 1 shows the baseline characteristics according to the use of glucosamine (non-users vs. users). Among all of the participants, 270,044 (54.45%) were female, and the mean age was 56.54 years. Overall, 94,498 (19.05%) reported habitual use of glucosamine supplement at baseline. Compared with non-users, glucosamine users were older, were more likely to be female, were of White ethnicity, were least deprived, had professional qualifications, had lower BMI, and had a higher prevalence of hypertension and arthritis at baseline. At the same time, glucosamine users tended to be never smokers, had a lower alcohol consumption, had a healthy diet, and reported more often use of vitamin and mineral supplements.

### Glucosamine use and dementia

During a median follow-up of 11 years, 6831 participants developed dementia (including 1790 AD and 923 VD). Table 2 shows the association between glucosamine use and risk of dementia. In comparison with glucosamine non-users, the incidence rate of dementia in those who reported use of glucosamine was 0.21 (95% CI: 0.19–0.22) per 1000 person-years. The incidence rate of AD was 0.06 (0.05–0.07) per 1000 person-years for glucosamine users, and the incidence rate of VD was 0.03 (0.02–0.03) per 1000 person-years for glucosamine users. In the age- and sex-adjusted models, we found an inverse association between glucosamine use and risk of dementia (HR = 0.84; 95% CI: 0.79–0.89). The magnitude of the association was slightly attenuated if the models were further adjusted for a series of confounders (HR = 0.87; 95% CI: 0.82–0.93). We also examined the association between glucosamine use and subtypes of dementia. Similarly, participants reported use of glucosamine had an 18% decreased risk of incident VD compared with non-users (HR = 0.82; 95% CI: 0.69–0.98). No significant association between glucosamine use and AD was found in the multivariable-adjusted analysis (HR = 0.97; 95% CI: 0.86–1.08).

**Table 2:** Association of habitual glucosamine use and risk of dementia

	Dementia		Alzheimer’s disease		Vascular dementia	
	Glucosamine non-users	Glucosamine users	Glucosamine non-users	Glucosamine users	Glucosamine non-users	Glucosamine users
**No. of events**	5418	1413	1361	429	754	169
**Incidence rate**^a^	0.20 (0.19–0.20)	0.21 (0.19–0.22)	0.05 (0.04–0.05)	0.06 (0.05–0.07)	0.03 (0.02–0.03)	0.03 (0.02–0.03)
**Hazard ratios (95% CI)**						
**Model 1**^**b**^	1 (Reference)	0.84 (0.79–0.89)	1 (Reference)	0.96 (0.86–1.07)	1 (Reference)	0.71 (0.60–0.84)
**Model 2**^**c**^	1 (Reference)	0.90 (0.84–0.95)	1 (Reference)	0.98 (0.88–1.10)	1 (Reference)	0.81 (0.69–0.97)
**Model 3**^**d**^	1 (Reference)	0.87 (0.82–0.93)	1 (Reference)	0.97 (0.86–1.08)	1 (Reference)	0.82 (0.69–0.98)

^a^Per 1000 person-years (95% CI)

^b^Model 1: Cox proportional hazards regression adjusted for age (timescale) and sex

^c^Model 2: Cox proportional hazards regression adjusted for age (timescale), sex, ethnicity, BMI, education attainment, Townsend deprivation index, smoking status, alcohol consumption, family history of dementia, hypertension, arthritis, total cholesterol, healthy diet, and *APOE ε*4 carrier.

^d^Model 3: Cox proportional hazards regression adjusted for age (timescale), sex, ethnicity, BMI, education attainment, Townsend deprivation index, smoking status, alcohol consumption, family history of dementia, hypertension, arthritis, total cholesterol, healthy diet, *APOE ε*4 carrier, mineral and vitamin supplements use, and aspirin use

### Interaction effect of glucosamine use and *APOE ε*4 genotype on dementia risk

We observed a joint effect of glucosamine use and *APOE ε4* genotype on risk of dementia (Table 3). Compared to the reference group (glucosamine users and *APOE ε4* non-carrier), the glucosamine non-users and *APOE ε4* carrier group (HR = 2.80, 95% CI: 2.58–3.04) was strongly associated with an increased risk of dementia. The adjusted HR of dementia risk for glucosamine users and APOE *ε4* carrier group was 2.75 (95% CI: 2.49–3.04). All indices of the additive interaction were statistically significant (AP = −0.072, 95% CI: −0.167, 0.024; RERI = −0.197, 95% CI: −0.451, 0.058; SI = 0.899, 95% CI: 0.784, 1.031).

**Table 3:** Interaction effect of glucosamine use and APOE *ε*4 genotype on the risk of dementia onset

	Risk of dementia by exposure, HR (95% CI)				Additive interaction (95% CI)			Multiplicative interaction (95% CI) ^**a**^
	Glucosamine users and APOE ***ε***4 non-carrier	Glucosamine users and APOE ***ε***4 carrier	Glucosamine non-users and APOE ***ε***4 non-carrier	Glucosamine non-users and APOE ***ε***4 carrier	AP	RERI	SI	
**Model 1**^**c**^	1 (Reference)	2.74 (2.48–3.03)	1.18 (1.10–1.28)	2.95 (2.72–3.19)	−0.142 (−0.641, 0.136)	−0.388 (−0.641, −0.136)	0.818 (0.718, 0.931)	1.25 (1.11–1.41) *P*<0.001
**Model 2**^**d**^	1 (Reference)	2.93 (2.63–3.27)	1.20 (1.10–1.31)	3.04 (2.78–3.32)	−0.105 (−0.206, −0.004)	−0.308 (−0.592, −0.023)	0.863 (0.754, 0.988)	1.22 (1.08–1.38) *P*=0.002
**Model 3**^**e**^	1 (Reference)	2.75 (2.49–3.04)	1.14 (1.05–1.24)	2.80 (2.58–3.04)	−0.072 (−0.167, 0.024)	−0.197 (−0.451, 0.058)	0.899 (0.784, 1.031)	1.26 (1.12–1.42) *P*<0.001
**Dementia subtype**^**b**^								
**AD**	1 (Reference)	4.07 (3.38–4.89)	0.95 (0.80–1.13)	3.83 (3.26–4.50)	0.069 (−0.063, 0.201)	0.280 (−0.271, 0.832)	1.101 (0.909, 1.333)	1.28 (1.01–1.61) *P*=0.039
**VD**	1 (Reference)	2.72 (2.03–3.63)	1.24 (0.99–1.56)	3.00 (2.38–3.78)	−0.193 (−1.073, 0.688)	−0.523 (−2.459, −1.412)	0.766 (0.234, 2.515)	1.30 (0.92–1.83) *P*=0.137

*Abbreviations*: *HR* Hazard ratio, *CI* Confidence interval, *AP* Attributable proportion due to interaction, *RERI* Relative excess risk due to interaction, *SI* Synergy index, *AD* Alzheimer’s disease, *VD* Vascular dementia

^a^Null hypothesis for each interaction is AP = 0, RERI = 0, SI = 1, and multiplicative interaction = 1

^b^Subgroup analysis for dementia subtype was based on Model 3

^c^Model 1: Cox proportional hazards regression adjusted for age (timescale) and sex

^d^Model 2: Cox proportional hazards regression adjusted for age (timescale), sex, ethnicity, BMI, education attainment, Townsend deprivation index, smoking status, alcohol consumption, family history of dementia, hypertension, arthritis, total cholesterol, and healthy diet

^e^Model 3: Cox proportional hazards regression adjusted for age (timescale), sex, ethnicity, BMI, education attainment, Townsend deprivation index, smoking status, alcohol consumption, family history of dementia, hypertension, arthritis, total cholesterol, healthy diet, mineral and vitamin supplements use, and aspirin use

Meanwhile, there were significant multiplicative interaction between glucosamine use and *APOE ε4* genotype (*P* for interaction = 0.0002). Stronger association between glucosamine use and decreased risk of dementia was observed in participants without *APOE ε4* carriers (HR = 0.81; 95% CI: 0.74–0.89) when compared to those with *APOE ε4* carriers (HR = 0.93; 95% CI: 0.86–1.02) (Supplementary Table 2).

### Subgroup analyses

We performed stratified analyses according to potential risk factors after imputation of missing values for covariates. With adjustment for age, sex, ethnicity, BMI, education attainment, Townsend deprivation index, smoking status, alcohol consumption, family history of dementia, hypertension, arthritis, total cholesterol, healthy diet, mineral and vitamin supplements use, and aspirin use, a lower HR between glucosamine use and risk of dementia was observed in participants with higher socioeconomic status; however, the effect modification was not statistically significant after multiple corrections (*P* for interaction = 0.018) (Supplementary Table 3). The association between glucosamine use and risk of dementia was not modified by other stratifying risk factors listed above, with all *P* values for interaction of > 0.05. Notably, we found that glucosamine use was significantly associated with a lower risk of dementia whether in participants who took mineral and vitamin supplements or not. Similar results were found in other lifestyle-related factors, including current smoking status, BMI, and healthy diet. In addition, the associations of glucosamine use with risk of AD and VD were not significantly modified by these risk factors (Supplementary Table 4).

### Role of T2D in the association between glucosamine use and dementia

**Fig. 1:**
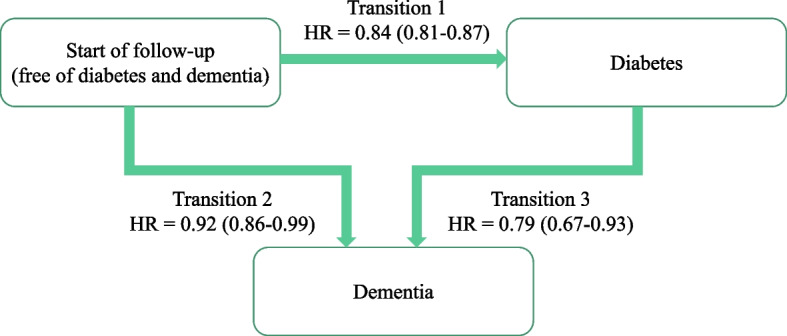
A multi-state model for roles of type 2 diabetes of the association between glucosamine use and dementia. Start of follow-up is a state in which participants free of type 2 diabetes and dementia (*N* = 485,106). Hazard ratios (HR) due to glucosamine use on the risk of transition from: (1) free of type 2 diabetes and dementia to incident type 2 diabetes (cases = 28,777); (2) free of type 2 diabetes and dementia to incident dementia (cases = 5,336); (3) type 2 diabetes to incident dementia (cases = 717). All HRs adjusted for age, sex, ethnicity, BMI, education attainment, Townsend deprivation index, smoking status, alcohol consumption, family history of dementia, hypertension, arthritis, total cholesterol, healthy diet, *APOE ε*4 carrier, mineral and vitamin supplements use, and aspirin use

**Fig. 2:**
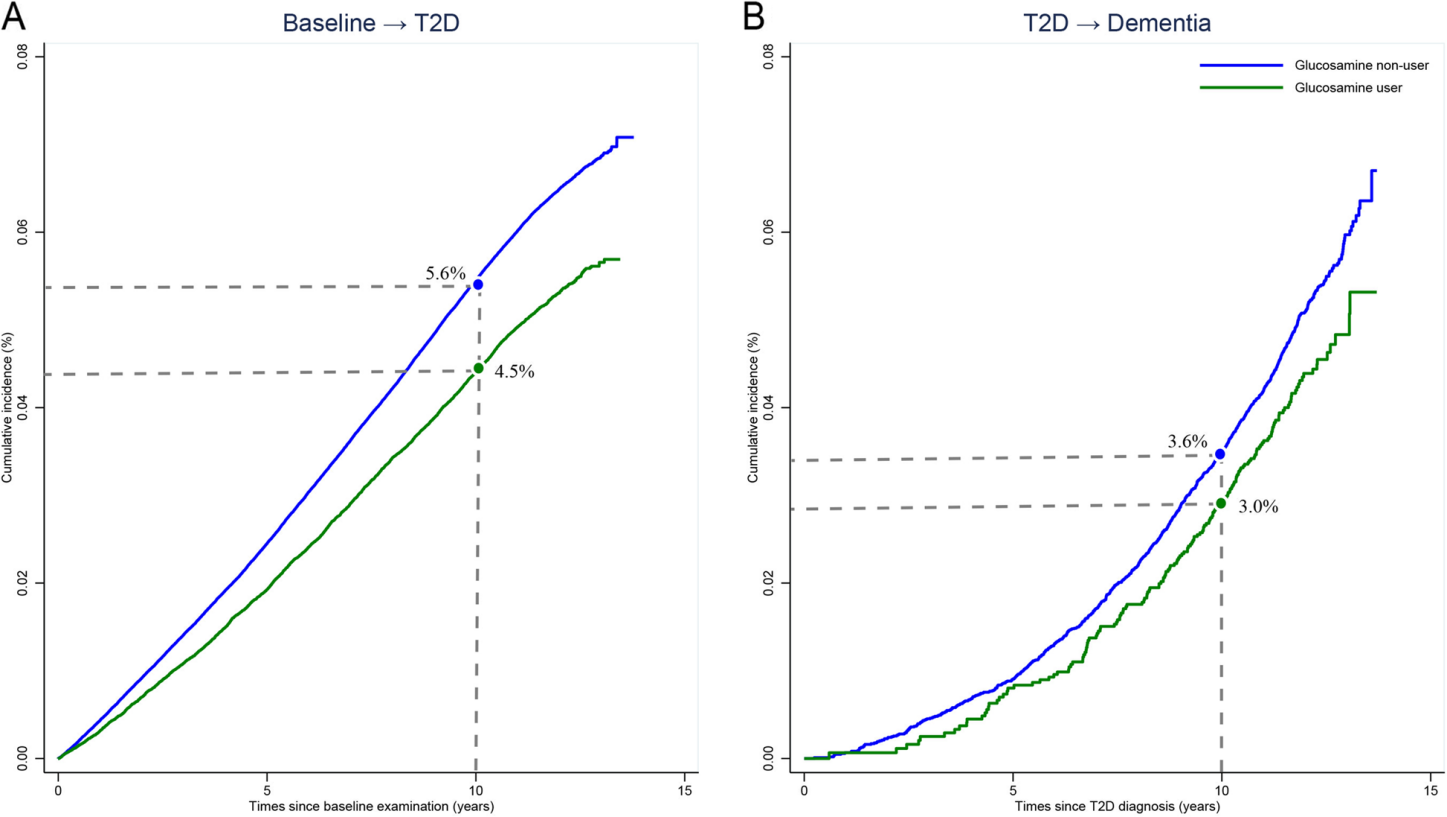
Cumulative incidence of type 2 diabetes and post-diabetes dementia. *Abbreviations*: *T2D* type 2 diabetes. The cumulative incidence was derived from Nelson-Aalen cumulative hazard function, and the cumulative incidence of T2D and post-T2D dementia at 10th year was calculated. **A** Cumulative incidence function of T2D for glucosamine non-users (blue) and glucosamine users (green). **B** Cumulative incidence function of post-diabetes dementia for glucosamine non-users (blue) and glucosamine users (green)

Figure 1 shows results from multi-state models in participants free of T2D and dementia. Glucosamine use was associated with a lower risk of incident T2D over the follow-up (Transition 1: HR = 0.84; 95% CI: 0.81–0.87). In those with T2D over the follow-up, glucosamine use was associated with a lower risk of dementia (Transition 3: HR = 0.79; 95% CI: 0.67–0.93). This suggested that the lower risk of dementia associated with glucosamine use and dementia could partly be explained by clinically T2D over the follow-up. The cumulative incidence of dementia on time since T2D diagnosis is shown in Fig. 2. Participants who reported without use of glucosamine during the follow-up had higher probability of developing dementia after T2D diagnosis. For example, in the tenth year after T2D diagnosis, the corresponding estimates were 3.6% for glucosamine non-users and 3.0% for users.

### Sensitivity analyses

Several sensitivity analyses were performed to support the robustness of our findings. Similar findings to the main analyses were found in the sensitivity analyses by excluding the participants who developed dementia events during the first 2 years of follow-up (Supplementary Table 5), performing the complete-case analyses and multiple imputation for missing covariates (Supplementary Table 6). The competing risk analysis also yielded consistent findings with those from Cox proportion hazards model (Supplementary Table 7). The *E*-values (rate ratios) of the point estimate and lower confidence bound for glucosamine-dementia association were 1.46 and 1.29. To nullify the observed association, an unmeasured confounding variable would need to be associated with glucosamine use and dementia by a risk ratio of at least 1.46, independent of the other confounding variables in the fully adjusted model.

## Discussion

In this large prospective cohort study of 495,942 UK adults, we found that glucosamine use was associated with a 10% lower risk of dementia, there were significant additive and multiplicative interactions between glucosamine use and *APOE ε*4 genotype. Moreover, the association was mediated by T2D, suggesting that the protective benefits of glucosamine use on risk of dementia might be partly explained by preventing or delaying the incidence of T2D over the follow-up.

Our current study found a decreased risk of dementia in glucosamine users from a perspective of epidemiology. The underlying mechanism of the association is poorly understood. It has been found that glucosamine is associated with decreased inflammation in cell culture studies [26]. Existing animal studies have also reported that the anti-inflammatory properties of glucosamine may play a preventive role in the pathophysiology of adverse health events [27–29]. A previous animal study revealed that glucosamine could mimic a low carbohydrate diet, which were characterized by decreasing glycolysis and improved amino acid catabolism [30]. This may also partly explain the anti-inflammatory effect of glucosamine because low carbohydrate diets were significantly associated with a lower risk of dementia as reported from a randomized clinical trial [31]. Other biological plausibility for the potential protective effect of glucosamine on dementia included its antioxidant activities in brain tissue. Glucosamine was involved into antioxidant activities by scavenging the superoxide and hydroxyl radicals and protecting the macromolecules. As oxidative stress had been consistently determined to link to increased dementia risk [32], the antioxidant properties of glucosamine may thus help with interpreting its potential anti-dementia mechanism. In a preclinical test, glucosamine can penetrate the blood-brain barrier and have a positive impact on the ability to complete cognitive tasks [33]. Glucosamine was also shown to improve spatial learning and memory in rats [34]. In small mammals and invertebrates, glucosamine promotes the generation of mitochondria, thereby providing energy for cells [30].

We found that the association between glucosamine use and dementia differed by *APOE* genotype. Stronger association between glucosamine use and decreased risk of dementia was observed in participants without *APOE ε*4 carriers. One of the possible explanations is that the *ε*4 allele of the apolipoprotein E gene is the strongest genetic risk factor for late-onset AD [35], and it has been identified as a risk factor for other dementia sub-type [36]. Moreover, the evidence from the Rotterdam study suggested that a healthy lifestyle can attenuate the risk of dementia [37]. A UK Biobank study found that a favorable lifestyle was associated with a lower risk of dementia, even in those who are at high genetic risk [38]. In our study, it is of great significance to find that glucosamine users were associated with a lower risk of dementia, regardless of adherence to a healthy lifestyle (such as a healthy diet, no current smoking, and normal BMI) or not. Moreover, previous studies have reported that glucosamine was often used in conjunction with other supplements [8], and our study consistently showed that glucosamine users tended to take vitamin and mineral supplements in combination than non-users. Similarly, we found that glucosamine use was associated with a lower risk of dementia, independent of mineral and vitamin supplement use.

We also observed that glucosamine user had a lower risk of subsequent dementia among those with developing T2D during the follow-up period. This means that the protective benefits of glucosamine use on risk of dementia might be partly explained by preventing or delaying the incidence of T2D over the follow-up. It has been suggested that T2D is an independent risk factor of all-cause dementia, including AD and VD [39, 40]. Prior longitudinal studies have ascertained the protective effects of glucosamine use on risk of incident T2D [12]. Suggested pathways that underpin the links between T2D and dementia may include systemic insulin resistance, and increased levels of circulating pro-inflammatory markers, which lead to defects in the insulin signaling pathway and changes in brain synaptic plasticity [41].

In analyses of secondary outcomes, we found a reverse association between glucosamine use and VD rather than AD, which may result from small sample size in the dementia subtype, and different mechanisms of dementia. The main pathophysiology of AD is beta-amyloid peptide deposition that leads to a cascade of neuronal cell apoptosis [42, 43]. However, VD occurs mainly through ischemia of the brain parenchyma caused by atherosclerotic disease. It is assumed that glucosamine use may exert on a beneficial effect against VD by influencing cardiovascular health. Future studies are warranted to elucidate the precise pathophysiological pathways from glucosamine to AD and VD.

To the best of our knowledge, the association between glucosamine use and dementia in such a large sample has not been reported. Besides large sample size, our study has the advantage of prospective design, longer follow-up time, and ability to adjust for potential confounders. Several sensitivity analyses also supported the robustness of our findings. Nevertheless, there are some unavoidable limitations of this study. First, the results we found were from an observational cohort study rather than a clinical trial, so conclusions regarding causality cannot be made. Second, dementia diagnoses in this study were obtained from electronic health records; the numbers of AD and VD are relatively small compared to the number of dementia cases, since the presence of large amount of other and unspecified dementia cases. We will have inevitably omitted undiagnosed conditions and less severe dementia cases that are largely dealt with in electronic health records. However, validation studies have suggested that these are reliable for ascertaining dementia, with a positive predictive value of 84.5% in UK Biobank when compared with expert clinical adjudication [44, 45]. Third, habitual glucosamine use was collected by self-reported at baseline, and information on form, duration, or dosage was not fully taken into account. Therefore, we could not explore the nature of potential associations between dosage and duration of glucosamine use with the defined outcomes. Finally, taking glucosamine could represent a healthy lifestyle. Although lifestyle-related factors were adjusted as well as *E*-values were calculated, it is difficult to disentangle the effects of a healthy lifestyle from the habitual glucosamine supplements. It is possible that unmeasured confounders and reverse causation remain in an observational study.

## Conclusions

To sum up, habitual glucosamine use was significantly associated with a lower risk of dementia, and the association was mediated by the incidence of T2D. Further studies should be conducted to further explore the potential biological plausibility and pharmacological pathways to explain the benefits use of glucosamine supplement.

## Supplementary Information

**Additional file 1: Supplementary Table 1.** List of ICD-10 codes for dementia. **Supplementary Table 2.** Association of habitual glucosamine use and risk of dementia stratified by *APOE ε*4 status. **Supplementary Table 3.** Association of habitual glucosamine use and risk of dementia stratified by potential risk factors. **Supplementary Table 4.** Associations of habitual glucosamine use and the risk of Alzheimer’s disease and vascular dementia stratified by potential risk factors. **Supplementary Table 5.** Association of glucosamine supplement use and risk of dementia after excluding participants with limited follow-up years (≤ 2 years). **Supplementary Table 6.** Association of glucosamine supplement use and risk of dementia in completed-case analysis (*n* = 448,043) and multiple imputation samples. **Supplementary Table 7.** Association of glucosamine supplement use and risk of dementia in compete risk models. **Supplementary Figure 1.** Flowchart of participant enrolment.

## Data Availability

The datasets used and/or analyzed during the current study are available from the corresponding author on reasonable request.

## Abbreviations

AD: Alzheimer’s disease
VD: Vascular dementia
HRs: Hazard ratios
CIs: Confidence intervals
T2D: Type 2 diabetes
CVD: Cardiovascular diseases
ICD-10: International Classification of Disease version 10
*APOE ε*4: Apolipoprotein E type 4
BMI: Body mass index
AP: Attributable proportion due to interaction
RERI: Relative excess risk due to interaction
SI: Synergy index
