# Supplementary material for: The role of type 2 diabetes in the association between habitual glucosamine use and dementia: a prospective cohort study
Source: Alzheimers Res Ther. 2022 Dec 13;14:184. doi: 10.1186/s13195-022-01137-x (PMC9746022; doi:10.1186/s13195-022-01137-x)
Supplement: Supplementary file 1 — Additional file 1: Supplementary Table 1. List of ICD-10 codes for dementia. Supplementary Table 2. Association of habitual glucosamine use and risk of dementia stratified by APOE ε4 status. Supplementary Table 3. Association of habitual glucosamine use and risk of dementia stratified by potential risk factors. Supplementary Table 4. Associations of habitual glucosamine use and the risk of Alzheimer’s disease and vascular dementia stratified by potential risk factors. Supplementary Table 5. Association of glucosamine supplement use and risk of dementia after excluding participants with limited follow-up years (≤ 2 years). Supplementary Table 6. Association of glucosamine supplement use and risk of dementia in completed-case analysis (n = 448,043) and multiple imputation samples. Supplementary Table 7. Association of glucosamine supplement use and risk of dementia in compete risk models. Supplementary Figure 1. Flowchart of participant enrolment. [file 13195_2022_1137_MOESM1_ESM.docx]

**Supplementary Materials**

**Supplementary Table 1:** List of ICD-10 codes for dementia.

**Supplementary Table 2:** Association of habitual glucosamine use and risk of dementia stratified by *APOE ε*4 status.

**Supplementary Table 3:** Association of habitual glucosamine use and risk of dementia stratified by potential risk factors.

**Supplementary Table 4:** Associations of habitual glucosamine use and the risk of Alzheimer’s disease and vascular dementia stratified by potential risk factors.

**Supplementary Table 5:** Association of glucosamine supplement use and risk of dementia after excluding participants with limited follow-up years (≤ 2 years).

**Supplementary Table 6:** Association of glucosamine supplement use and risk of dementia in completed-case analysis (n= 448,043) and multiple imputation samples**.**

**Supplementary Table 7:** Association of glucosamine supplement use and risk of dementia in compete risk models.

**Supplementary Figure 1:** Flowchart of participant enrolment.

| **Supplementary Table 1. List of ICD-10 codes for dementia.** | | |
| --- | --- | --- |
| **Code** | **Term** | **Subtype** |
| F00 | Dementia in Alzheimer's disease | AD |
| F000 | Dementia in Alzheimer's disease with early onset | AD |
| F001 | Dementia in Alzheimer's disease with late onset | AD |
| F002 | Dementia in Alzheimer's disease, atypical or mixed type | AD |
| F009 | Dementia in Alzheimer's disease, unspecified | AD |
| G30 | Alzheimer’s disease | AD |
| G300 | Alzheimer’s disease with early onset | AD |
| G301 | Alzheimer’s disease with late onset | AD |
| G308 | Other Alzheimer’s disease | AD |
| G309 | Alzheimer’s disease, unspecified | AD |
| F01 | Vascular dementia | VD |
| F010 | Vascular dementia of acute onset | VD |
| F011 | Multi-infarct dementia | VD |
| F012 | Subcortical vascular dementia | VD |
| F013 | Mixed cortical and subcortical vascular dementia | VD |
| F018 | Other vascular dementia | VD |
| F019 | Vascular dementia, unspecified | VD |
| F02 | Dementia in other diseases classified elsewhere | N |
| F020 | Dementia in Pick's disease | FTD |
| G310 | Circumscribed brain atrophy | FTD |
| F021 | Dementia in Creutzfeldt-Jakob disease | O |
| F022 | Dementia in Huntington's disease | O |
| F023 | Dementia in Parkinson's disease | O |
| F024 | Dementia in human immunodeficiency virus [HIV] disease | O |
| F028 | Dementia in other specified diseases classified elsewhere | N |
| F03 | Unspecified dementia | N |
| G311 | Senile degeneration of brain, not elsewhere classified | N |
| G312 | Degeneration of nervous system due to alcohol | N |
| G318 | Other specified degenerative diseases of nervous system | N |
| G319 | Degenerative disease of nervous system, unspecified | N |
| *Abbreviations*: *AD* Alzheimer’s disease, *VD* vascular dementia, *FTD* frontotemporal dementia, *O* other dementia subtype, *N* no subtype specified. | | |

| **Supplementary Table 2. Association of habitual glucosamine use and risk of dementia stratified by APOE *ε4* status.** | | | |
| --- | --- | --- | --- |
| **Subgroup** | **Total** | **Hazard ratios (95% CI)** | P for interaction |
| ***APOE ε4* carrier** |  |  | 0.0002 |
| No | 358,161 | 0.81 (0.74-0.89) |  |
| Yes | 137,781 | 0.93 (0.86-1.02) |  |
| Results were adjusted for age, sex, ethnicity, BMI, education attainment, Townsend deprivation index, smoking status, alcohol consumption, family history of dementia, hypertension, arthritis, total cholesterol, healthy diet, mineral and vitamin supplements use, and aspirin use. | | | |

| **Supplementary Table 3. Association of habitual glucosamine use and risk of dementia stratified by potential risk factors.** | | | | |
| --- | --- | --- | --- | --- |
| **Subgroup** | | **Total** | **Hazard ratios (95% CI)** | P for interaction |
| **Age** | |  |  | 0.076 |
|  | < 60 years | 280,918 | 0.76 (0.63-0.91) |  |
|  | ≥ 60 years | 215,024 | 0.89 (0.84-0.95) |  |
| **Sex** | |  |  | 0.822 |
|  | Female | 270,044 | 0.87 (0.80-0.94) |  |
|  | Male | 225,898 | 0.89 (0.81-0.97) |  |
| **Ethnicity** | |  |  | 0.480 |
|  | White | 467,865 | 0.88 (0.82-0.94) |  |
|  | Non-white | 28,077 | 0.80 (0.58-1.10) |  |
| **Education attainment** | |  |  | 0.194 |
|  | College or university degree | 335,150 | 0.85 (0.80-0.92) |  |
|  | Professional qualifications | 160,792 | 0.90 (0.79-1.02) |  |
| **Townsend deprivation index** | |  |  | 0.018 |
|  | < median | 298,167 | 0.90 (0.83-0.98) |  |
|  | ≥ median | 197,774 | 0.80 (0.73-0.89) |  |
| **Body mass index** | |  |  | 0.051 |
|  | < 30 kg/m2 | 374,028 | 0.90 (0.84-0.97) |  |
|  | ≥ 30 kg/m2 | 121,914 | 0.81 (0.72-0.92) |  |
| **Current smoking** | |  |  | 0.073 |
|  | No | 443,810 | 0.89 (0.83-0.95) |  |
|  | Yes | 52,132 | 0.75 (0.60-0.94) |  |
| Moderate alcohol intake † | |  |  | 0.983 |
|  | No | 126,906 | 0.92 (0.81-1.05) |  |
|  | Yes | 369,036 | 0.87 (0.81-0.93) |  |
| **Healthy diet** | |  |  | 0.690 |
|  | No | 246,838 | 0.88 (0.79-0.96) |  |
|  | Yes | 249,104 | 0.87 (0.80-0.94) |  |
| **Family history of dementia** | |  |  | 0.459 |
|  | No | 430,781 | 0.88 (0.82-0.94) |  |
|  | Yes | 65,161 | 0.87 (0.76-1.00) |  |
| Hypercholesterolemia ‡ | |  |  | 0.467 |
|  | No | 382,286 | 0.88 (0.82-0.95) |  |
|  | Yes | 113,656 | 0.82 (0.72-0.94) |  |
| **Hypertension** | |  |  | 0.149 |
|  | No | 150,216 | 0.81 (0.71-0.93) |  |
|  | Yes | 345,726 | 0.89 (0.83-0.96) |  |
| Arthritis | |  |  | 0.603 |
|  | No | 443,728 | 0.89 (0.83-0.95) |  |
|  | Yes | 52,214 | 0.84 (0.74-0.95) |  |
| **Mineral and vitamin use** | |  |  | 0.956 |
|  | No | 338,492 | 0.88 (0.81-0.96) |  |
|  | Yes | 157,450 | 0.87 (0.79-0.95) |  |
| **Aspirin use** | |  |  | 0.100 |
|  | No | 426,624 | 0.90 (0.84-0.97) |  |
|  | Yes | 69,318 | 0.80 (0.71-0.91) |  |
| Results were adjusted for age, sex, ethnicity, BMI, education attainment, Townsend deprivation index, smoking status, alcohol consumption, family history of dementia, hypertension, arthritis, total cholesterol, healthy diet, *APOE* *ε*4 carrier, mineral and vitamin supplements use, and aspirin use. | | | | |

| **Supplementary Table 4. Associations of habitual glucosamine use and the risk of Alzheimer’s disease and vascular dementia stratified by potential risk factors.** | | | | | | |
| --- | --- | --- | --- | --- | --- | --- |
| **Subgroup** | | **Alzheimer’s disease** | |  | **Vascular dementia** | |
|  |  | **Hazard ratios  (95% CI)** | ***P*** for interaction |  | **Hazard ratios  (95% CI)** | ***P*** for interaction |
| **Age** | |  | 0.079 |  |  | 0.868 |
|  | < 60 years | 0.67 (0.44-1.02) |  |  | 0.85 (0.46-1.57) |  |
|  | ≥ 60 years | 1.00 (0.98-1.23) |  |  | 0.82 (0.68-0.98) |  |
| **Sex** | |  | 0.153 |  |  | 0.531 |
|  | Female | 0.92 (0.79-1.07) |  |  | 0.85 (0.66-1.08) |  |
|  | Male | 1.04 (0.87-1.24) |  |  | 0.79 (0.62-1.03) |  |
| **Ethnicity** | |  | 0.414 |  |  | 0.320 |
|  | White | 0.97 (0.87-1.09) |  |  | 0.83 (0.70-1.00) |  |
|  | Non-white | 0.73 (0.37-1.44) |  |  | 0.54 (0.18-1.56) |  |
| **Education attainment** | |  | 0.132 |  |  | 0.859 |
|  | College or university degree | 0.91 (0.80-1.03) |  |  | 0.84 (0.70-1.02) |  |
|  | Professional qualifications | 1.15 (0.90-1.48) |  |  | 0.68 (0.45-1.02) |  |
| **Townsend deprivation index** | |  | 0.813 |  |  | 0.552 |
|  | < median | 0.99 (0.86-1.14) |  |  | 0.76 (0.60-0.96) |  |
|  | ≥ median | 0.92 (0.76-1.11) |  |  | 0.88 (0.68-1.16) |  |
| **Body mass index** | |  | 0.229 |  |  | 0.557 |
|  | < 30 kg/m^2^ | 1.00 (0.88-1.14) |  |  | 0.76 (0.61-0.95) |  |
|  | ≥ 30 kg/m^2^ | 0.87 (0.68-1.10) |  |  | 0.96 (0.76-1.28) |  |
| **Current smoking** | |  | 0.803 |  |  | 0.408 |
|  | No | 0.98 (0.87-1.10) |  |  | 0.83 (0.69-1.00) |  |
|  | Yes | 0.81 (0.50-1.31) |  |  | 0.70 (0.37-1.34) |  |
| Moderate alcohol intake † | |  | 0.361 |  |  | 0.414 |
|  | No | 1.03 (0.81-1.32) |  |  | 1.08 (0.74-1.55) |  |
|  | Yes | 0.95 (0.83-1.08) |  |  | 0.76 (0.63-0.93) |  |
| **Healthy diet** | |  | 0.053 |  |  | 0.624 |
|  | No | 1.09 (0.91-1.31) |  |  | 0.81 (0.62-1.08) |  |
|  | Yes | 0.89 (0.77-1.03) |  |  | 0.82 (0.65-1.03) |  |
| **Family history of dementia** | |  | 0.631 |  |  | 0.819 |
|  | No | 0.96 (0.84-1.09) |  |  | 0.82 (0.68-1.00) |  |
|  | Yes | 0.99 (0.79-1.23) |  |  | 0.81 (0.55-1.20) |  |
| Hypercholesterolemia ‡ | |  | 0.366 |  |  | 0.287 |
|  | No | 1.00 (0.88-1.14) |  |  | 0.79 (0.65-0.97) |  |
|  | Yes | 0.87 (0.69-1.09) |  |  | 0.88 (0.60-1.28) |  |
| **Hypertension** | |  | 0.941 |  |  | 0.114 |
|  | No | 0.99 (0.76-1.28) |  |  | 0.65 (0.43-0.99) |  |
|  | Yes | 0.96 (0.85-1.09) |  |  | 0.87 (0.71-1.05) |  |
| Arthritis | |  | 0.040 |  |  | 0.014 |
|  | No | 1.03 (0.90-1.18) |  |  | 0.72 (0.59-0.89) |  |
|  | Yes | 0.81 (0.64-1.03) |  |  | 1.18 (0.84-1.66) |  |
| **Mineral and vitamin use** | |  | 0.837 |  |  | 0.523 |
|  | No | 0.95 (0.81-1.12) |  |  | 0.88 (0.69-1.11) |  |
|  | Yes | 0.98 (0.83-1.16) |  |  | 0.75 (0.58-0.98) |  |
| **Aspirin use** | |  | 0.241 |  |  | 0.231 |
|  | No | 0.99 (0.87-1.12) |  |  | 0.76 (0.61-0.96) |  |
|  | Yes | 0.89 (0.70-1.14) |  |  | 0.91 (0.69-1.21) |  |
| Results were adjusted for age, sex, ethnicity, BMI, education attainment, Townsend deprivation index, smoking status, alcohol consumption, family history of dementia, hypertension, arthritis, total cholesterol, healthy diet, *APOE ε*4 carrier, mineral and vitamin supplements use, and aspirin use. | | | | | | |

| **Supplementary Table 5. Association of glucosamine supplement use and risk of dementia after excluding participants with limited follow-up years (≤ 2 years).** | | | | | | |
| --- | --- | --- | --- | --- | --- | --- |
|  | **Dementia** | | **Alzheimer’s disease** | | **Vascular dementia** | |
|  | **Glucosamine non-users** | **Glucosamine users** | **Glucosamine non-users** | **Glucosamine users** | **Glucosamine non-users** | **Glucosamine users** |
| **No. of events** | 5,255 | 1,387 | 1,348 | 429 | 737 | 167 |
| **Hazard ratios**  **(95% CI)** |  |  |  |  |  |  |
| **Model 1 *** | 1 (Reference) | 0.84 (0.79-0.89) | 1 (Reference) | 0.97 (0.87-1.08) | 1 (Reference) | 0.72 (0.61-0.85) |
| **Model 2 †** | 1 (Reference) | 0.90 (0.85-0.95) | 1 (Reference) | 0.99 (0.89-1.11) | 1 (Reference) | 0.82 (0.69-0.97) |
| **Model 3 ‡** | 1 (Reference) | 0.88 (0.83-0.94) | 1 (Reference) | 0.97 (0.87-1.09) | 1 (Reference) | 0.83 (0.69-0.99) |
| * Model 1: Cox proportional hazards regression adjusted for age (timescale) and sex.  † Model 2: Cox proportional hazards regression adjusted for age (timescale), sex, ethnicity, BMI, education attainment, Townsend deprivation index, smoking status, alcohol consumption, family history of dementia, hypertension, arthritis, total cholesterol, healthy diet, and *APOE* *ε*4 carrier.  **‡** Model 3: Cox proportional hazards regression adjusted for age (timescale), sex, ethnicity, BMI, education attainment, Townsend deprivation index, smoking status, alcohol consumption, family history of dementia, hypertension, arthritis, total cholesterol, healthy diet, *APOE* *ε*4 carrier, mineral and vitamin supplements use, and aspirin use. | | | | | | |

| **Supplementary Table 6. Association of glucosamine supplement use and risk of dementia in completed-case analysis (n= 448,043) and multiple imputation samples.** | | | | | | |
| --- | --- | --- | --- | --- | --- | --- |
|  | **Completed-case analysis** | | | **Multiple imputation** | | |
|  | **Model 1** * | **Model 2** † | **Model 3 ‡** | **Model 1** * | **Model 2** † | **Model 3 ‡** |
| **Dementia** |  |  |  |  |  |  |
| Glucosamine non-users | 1 (Reference) | 1 (Reference) | 1 (Reference) | 1 (Reference) | 1 (Reference) | 1 (Reference) |
| Glucosamine users | 0.83 (0.78-0.88) | 0.88 (0.83-0.94) | 0.86 (0.80-0.91) | 0.84 (0.79-0.89) | 0.89 (0.84-0.95) | 0.87 (0.82-0.93) |
| **Alzheimer’s disease** |  |  |  |  |  |  |
| Glucosamine non-users | 1 (Reference) | 1 (Reference) | 1 (Reference) | 1 (Reference) | 1 (Reference) | 1 (Reference) |
| Glucosamine users | 0.95 (0.85-1.07) | 0.98 (0.87-1.10) | 0.96 (0.85-1.08) | 0.97 (0.87-1.08) | 0.99 (0.88-1.10) | 0.97 (0.87-1.09) |
| **Vascular disease** |  |  |  |  |  |  |
| Glucosamine non-users | 1 (Reference) | 1 (Reference) | 1 (Reference) | 1 (Reference) | 1 (Reference) | 1 (Reference) |
| Glucosamine users | 0.68 (0.56-0.81) | 0.77 (0.64-0.92) | 0.77 (0.64-0.93) | 0.71 (0.60-0.84) | 0.81 (0.68-0.96) | 0.82 (0.68-0.97) |
| * Model 1: Cox proportional hazards regression adjusted for age (timescale) and sex. | | | | | | |
| † Model 2: Cox proportional hazards regression adjusted for age (timescale), sex, ethnicity, BMI, education attainment, Townsend deprivation index, smoking status, alcohol consumption, family history of dementia, hypertension, arthritis, total cholesterol, healthy diet, *APOE* *ε*4 carrier. | | | | | | |
| **‡** Model 3: Cox proportional hazards regression adjusted for age (timescale), sex, ethnicity, BMI, education attainment, Townsend deprivation index, smoking status, alcohol consumption, family history of dementia, hypertension, arthritis, total cholesterol, healthy diet, *APOE ε*4 carrier, mineral and vitamin supplements use, and aspirin use. | | | | | | |

| **Supplementary Table 7. Association of glucosamine supplement use and risk of dementia in compete risk models.** | | | |
| --- | --- | --- | --- |
|  | **Model 1** * | **Model 2** † | **Model 3 ‡** |
| **Dementia** |  |  |  |
| Glucosamine non-users | 1 (Reference) | 1 (Reference) | 1 (Reference) |
| Glucosamine users | 0.87 (0.82-0.92) | 0.92 (0.87-0.98) | 0.91 (0.85-0.96) |
| **Alzheimer’s disease** |  |  |  |
| Glucosamine non-users | 1 (Reference) | 1 (Reference) | 1 (Reference) |
| Glucosamine users | 1.00 (0.90-1.12) | 1.02 (0.91-1.14) | 1.00 (0.89-1.12) |
| **Vascular disease** |  |  |  |
| Glucosamine non-users | 1 (Reference) | 1 (Reference) | 1 (Reference) |
| Glucosamine users | 0.75 (0.63-0.88) | 0.84 (0.71-1.00) | 0.85 (0.72-1.02) |
| * Model 1: Cox proportional hazards regression adjusted for age (timescale) and sex. | | | |
| † Model 2: Cox proportional hazards regression adjusted for age (timescale), sex, ethnicity, BMI, education attainment, Townsend deprivation index, smoking status, alcohol consumption, family history of dementia, hypertension, arthritis, total cholesterol, healthy diet, *APOE ε*4 carrier. | | | |
| **‡** Model 3: Cox proportional hazards regression adjusted for age (timescale), sex, ethnicity, BMI, education attainment, Townsend deprivation index, smoking status, alcohol consumption, family history of dementia, hypertension, arthritis, total cholesterol, healthy diet, *APOE ε*4 carrier, mineral and vitamin supplements use, and aspirin use. | | | |

**Supplementary Figure 1. Flowchart of participant enrolment.**
